# Identification of glucocorticoid-induced leucine zipper as a key regulator of tumor cell proliferation in epithelial ovarian cancer

**DOI:** 10.1186/1476-4598-8-83

**Published:** 2009-10-08

**Authors:** Nassima Redjimi, Françoise Gaudin, Cyril Touboul, Dominique Emilie, Marc Pallardy, Armelle Biola-Vidamment, Hervé Fernandez, Sophie Prévot, Karl Balabanian, Véronique Machelon

**Affiliations:** 1 UMR-S 764, INSERM/Université Paris-Sud, Clamart, France; 2 Service de Microbiologie - Immunologie Biologique, Assistance Publique-Hôpitaux de Paris-Hôpital Antoine Béclère, Clamart, France; 3 Service d'Anatomie et de Cytologie Pathologiques, Assistance Publique-Hôpitaux de Paris/Hôpital Antoine Béclère, Clamart, France; 4 Service de Gynécologie-Obstétrique et de Médecine de la Reproduction, Assistance Publique-Hôpitaux de Paris/Hôpital Antoine Béclère, Clamart, France; 5 UMR-S 749, INSERM/Université Paris-Sud, Chatenay-Malabry, France

## Abstract

**Background:**

Little is known about the molecules that contribute to tumor progression of epithelial ovarian cancer (EOC), currently a leading cause of mortality from gynecological malignancies. Glucocorticoid-Induced Leucine Zipper (GILZ), an intracellular protein widely expressed in immune tissues, has been reported in epithelial tissues and controls some of key signaling pathways involved in tumorigenesis. However, there has been no report on GILZ in EOC up to now. The objectives of the current study were to examine the expression of GILZ in EOC and its effect on tumor cell proliferation.

**Results:**

GILZ expression was measured by immunohistochemical staining in tissue sections from 3 normal ovaries, 7 benign EOC and 50 invasive EOC. GILZ was not detected on the surface epithelium of normal ovaries and benign tumors. In contrast, it was expressed in the cytoplasm of tumor cells in 80% EOC specimens. GILZ immunostaining scores correlated positively to the proliferation marker Ki-67 (Spearman test in univariate analysis, *P *< 0.00001, r = 0.56). They were also higher in tumor cells containing large amounts of phosphorylated protein kinase B (p-AKT) (unpaired t test, *P *< 0.0001). To assess the effect of GILZ on proliferation and AKT activation, we used the BG-1 cell line derived from ovarian tumor cells as a cellular model. GILZ expression was either enhanced by stable transfection or decreased by the use of small interfering (si) RNA targeting GILZ. We found that GILZ increased cell proliferation, phospho-AKT cellular content and AKT kinase activity. Further, GILZ upregulated cyclin D1 and phosphorylated retinoblastoma (p-Rb), downregulated cyclin-dependent kinase inhibitor p21, and promoted the entry into S phase of cell cycle.

**Conclusion:**

The present study is the first to identify GILZ as a molecule produced by ovarian cancer cells that promotes cell cycle progression and proliferation. Our findings clearly indicate that GILZ activates AKT, a crucial signaling molecule in tumorigenesis. GILZ thus appears as a potential key molecule in EOC.

## Background

Epithelial ovarian cancer (EOC) accounts for nearly 90% of ovarian malignant tumors [1,2]. Early stage ovarian carcinoma is silent in nature and therefore these carcinoma often expand into the peritoneal cavity and metastasize to the omentum before diagnosis. Consequently, treatment is particularly challenging and this malignancy is a leading cause of death among gynecological malignancies in developed countries [3]. The prognosis for patients with ovarian carcinoma is determined by conventional criteria, including tumor stage, histological type, and grade. Indeed, there is also a need to identify molecular markers that drive ovarian tumor progression, one of the least determined process in cancer research, to offer novel, targeted, biological therapy [4].

Glucocorticoid-Induced Leucine Zipper (GILZ) is a small leucine zipper protein of 17 kDa and a member of the TSC22D (Transforming Growth Factor1 Stimulated Clone 22 Domain) family of proteins also known as TSC22D3. GILZ was discovered as a dexamethasone-induced transcript in murine thymocytes [5]. It is widely expressed in immune tissues and has also been reported in epithelial tissues often associated to a hormonal background. It is rapidly induced by glucocorticoids in T lymphocytes [6-8], macrophages, dendritic cells and mast cells [9-11]. GILZ expression in the anterior pituitary during embryonic development in the chick is consistent with regulation by corticosteroids [12]; in the kidney cortical collecting duct, GILZ is induced by aldosterone [13]; and in human cervical adenocarcinoma HeLa cells, GILZ expression is controlled by estradiol [14].

GILZ interferes with Raf-1, nuclear factor-kB (NF-kB), AP-1 and FoxO forkhead transcription factor FoxO3 [15,8,17], all are key signaling molecules important for tumorigenesis [18]. There have, however, been few studies of GILZ in cancer. GILZ has been reported in multiple myeloma, in lymphoblastic leukemia and in human osteosarcoma cells [19-22]. Most relevant work has been in cell lines and very few data from human tumor specimens are available. To our knowledge, there is no report on GILZ in EOC. We therefore investigated GILZ expression and function in these malignant tumors. Our findings are supported by parallel and complementary data accumulated in tumor specimens and in the BG-1 cellular model. We report evidence that GILZ, an intracellular factor not previously described in EOC, plays a pivotal role in tumor cell proliferation.

## results

### GILZ detection in human ovarian tumor samples

**Figure 1:**
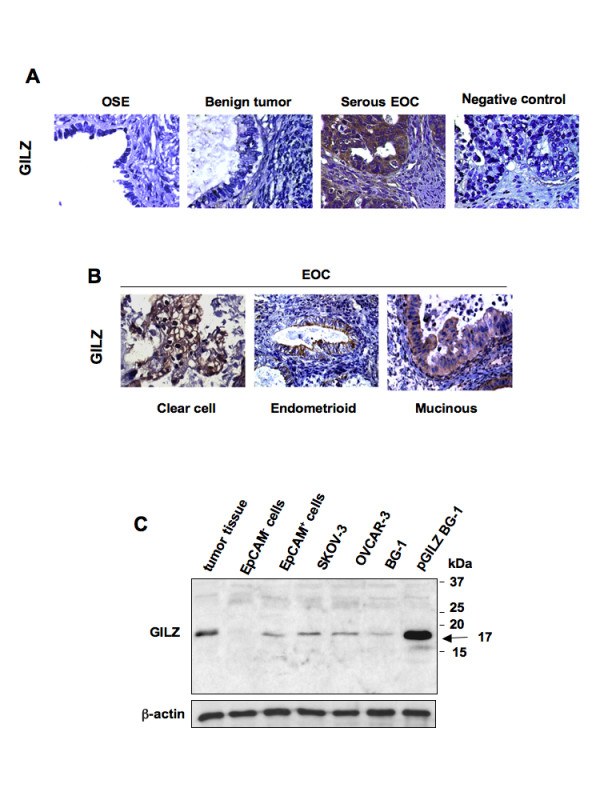
**GILZ detection in epithelial ovarian cancer (EOC)**. (A) GILZ immunostaining in ovarian surface epithelium of normal ovaries (OSE), benign tumors, invasive serous ovarian carcinoma. Negative control was done without primary Ab. Original magnification, ×63. (B) Cytoplasmic GILZ immunostaining in clear cell, endometrioid and mucinous EOC. Original magnification, ×40. (C) GILZ immunoblots of total protein lysates from ovarian cancer tissue (representative data from several frozen tumor specimens), malignant ascites processed by the autoMacs procedure to separate EpCAM+ cells identified as epithelial cells from EpCAM- cells, from ovarian cancer cell lines SKOV-3, OVCAR-3 and BG-1. BG-1 cells stably transfected with *GILZ*-encoding vector pcDNA3-GILZ (pGILZ) was used as positive control (17 kDa). Actin levels are shown for normalization. Data are representative of three different experiments.

GILZ expression was assessed by immunohistochemical staining of sections isolated from three normal ovaries, seven benign EOC and 50 invasive EOC. GILZ was not detected on the surface epithelium of normal ovaries and in benign tumors. In contrast, among the invasive ovarian cancers, 40 (80%) expressed GILZ. GILZ immunoreactivity was detected in the four main histological subtypes, serous, clear cell, endometrioid and mucinous tumors. It was clearly confined to the cytoplasm of tumor cells and was weak in, or absent from the tumor stroma (Figure 1A and 1B). Using the same antibody, we detected GILZ protein by western blot. GILZ was revealed at 17 kDa, which is the size of the protein described by Ricardi and co-workers in 1997 [5], in BG-1 cells transfected with the *GILZ*-encoding vector pcDNA3-GILZ (pGILZ BG-1 cells used as positive control), in epithelial cells from malignant ascites and in ovarian tumor samples for which frozen tissues were available, confirming the staining data (Figure 1C). Interestingly, the non-epithelial cells from malignant ascites do not express GILZ. GILZ 17 kDa protein was also detected in ovarian cancer cell lines SKOV-3, OVCAR-3 and BG-1; it was less abundant in BG-1 cell line than in SKOV-3 and OVCAR-3 (Figure 1C). BG-1 thus appeared as the best-fitted cellular model for processing up and down regulation of GILZ.

### GILZ expression correlates with tumor cell proliferation and with the expression levels of phospho-AKT in EOC

**Figure 2:**
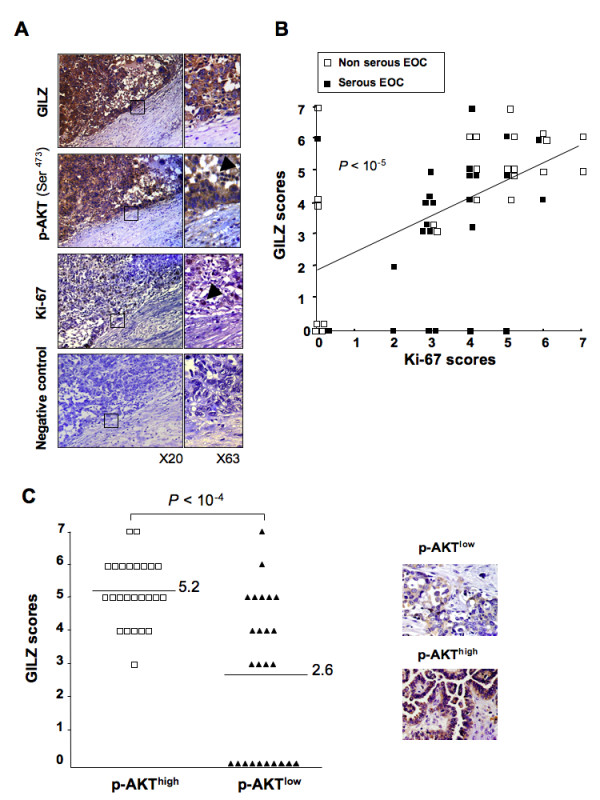
**GILZ expression correlates with p-AKT levels and with cell proliferation in epithelial ovarian cancer (EOC)**. (A) Serial sections of tumors with staining for p-AKT, GILZ and Ki-67. Negative control was done without primary Ab. Original magnifications, ×20 and ×63. Pictures show cytoplasmic staining for GILZ and nuclear staining for Ki-67; p-AKT staining is mostly in the cytoplasm with some additional nuclear staining (arrow). (B) GILZ and Ki-67 final scores were positively correlated (Spearman test in univariate analysis, *P *< 0.00001, r = 0.56). (C) GILZ scores for p-AKT^high ^and p-AKT^low ^groups (the difference was significant at *P *< 0.05, Student's t test). Insets: high and low p-AKT immunoreactivity is shown on representative micrographs.

GILZ immunostaining was unevenly distributed in tumor cells, from no detectable staining to strong immunoreactivity. We asked if differences in GILZ expression levels are related to the expression of two markers, the proliferation marker Ki-67 used in routine diagnostics [23] and p-AKT used to characterize malignant ovarian tumors [24]. Hyperactivation of AKT is frequently observed in ovarian neoplasms and is related to the control of cell proliferation in EOC [25,26]. Immunoreactivity of GILZ, Ki-67 and p-AKT was measured on serial sections of EOC (Figure 2A). GILZ and Ki-67 immunostainings were scored on a seven-point scale based on the staining intensity and the extent of staining. GILZ and Ki-67 expression scores were significantly correlated in the entire cohort (Spearman test in univariate analysis, *P *< 0.00001, r = 0.56) (Figure 2B). They were still correlated in serous carcinoma and non serous carcinoma as well (Spearman test in univariate analysis, *P *< 0.001, r = 0.61, n = 26 serous carcinoma, *P *< 0.02, r = 0.49, n = 24 non serous carcinoma). The expression of p-AKT in tumor cells was mostly cytoplasmic, although some nuclear staining was also detected (Figure 2A). Both nuclear and cytoplasmic staining patterns were considered to assess p-AKT immunoreactivity, scored as high or low. GILZ expression scores were significantly higher in p-AKT^high ^specimens (mean ± SE = 5.2 ± 0.2, n = 25) than in p-AKT^low ^specimens (mean ± SE = 2.6 ± 0.4, n = 25), (unpaired t test, *P *< 0.0001) (Figure 2C).

After applying a single cut-off on the entire cohort for identification of GILZ^high ^(scores 5-7, n = 24) and GILZ^low ^(scores 0-4, n = 26) cases, we found that high GILZ scores are associated with higher p-AKT staining and Ki-67 indexes (Fisher test, *P *< 0.0002 and *P *< 0.0008), (Table 1). In contrast, age at diagnosis and distribution of histological subtypes did not differ between the two groups (Table 1).

**Table 1:** GILZ immunostaining scores versus Ki-67 proliferation index and p-AKT immunostaining in ovarian carcinoma tissues (n = 50)

	**Patient number**		**Fisher test** **Statistical significance^a^**
	GILZ^low^ scores (0-4)	GILZ^high^ scores (5-7)	
			
All carcinomas	26	24	
			
Age at diagnosis			
< 60 years	13	13	ns
> 60 years	13	11	
			
Histological types			
Serous	13	13	ns
Non serous	13	11	
			
Clear cells	3	2	ns
Mucinous	5	4	
Endometrioid	4	4	
Undifferentiated	1	1	
			
Ki-67 Immuno-staining			
low scores (0-4)	23 (88%)	10 (42%)	*P *< 0.0008
high scores (5-7)	3 (12%)	14 (58%)	
			
Phospho-AKT immuno-staining			
low	20 (77%)	5 (21%)	*P *< 0.0002
high	6 (23%)	19 (79%)	

All these observations suggest that GILZ expression may regulate cell proliferation and AKT phosphorylation in EOC. To assess this hypothesis and to provide further biological evidence to support immunohistochemical data, we performed in vitro experiments using the BG-1 cell line as a cellular model.

### Overexpression of GILZ increases proliferation and AKT phosphorylation in BG-1 cells

**Figure 3:**
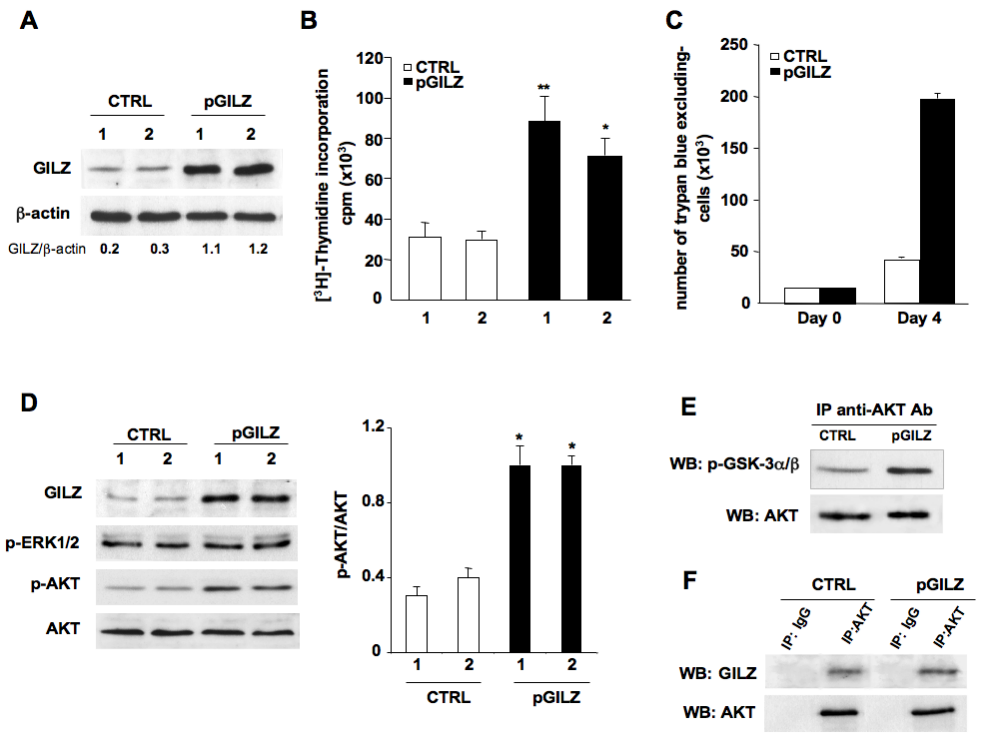
**Up regulation of GILZ increases cell proliferation and AKT activation**. (A) Total protein lysates analyzed by western bloting with anti-GILZ Ab in two CTRL and pGILZ clones. (B) Cell proliferation measured by [^3^H]-thymidine incorporation in two CTRL and pGILZ clones. Means ± SD of three independent experiments. Statistics used Kruskall Wallis test, *P < 0.05, **P < 0.01. (C) Number of trypan blue-excluding cells among CTRL and pGILZ clones seeded at 15 × 10^3 ^cells/well and cultured for 4 days. Means of triplicates of one representative experiment of three; error bars represent SE. (D) Left: total protein lysates from CTRL or pGILZ clones cultured overnight without serum analyzed by western blotting using specific Abs. Right: p-AKT signal quantified by densitometric analysis and normalized to total AKT. Mean ± SE of three experiments, *P < 0.05, unpaired Student's t test. (E) Total lysates from CTRL or pGILZ were immunoprecipitated (IP) using an anti-AKT Ab, and then incubated with GSK-3-fusion protein in an *in vitro *kinase assay. GSK-3 phosphorylation was assessed by western blot (WB) with phospho-specific GSK-3 Ab. Total AKT was used for normalization. (F) Total protein lysates from BG-1 cells were immnunoprecipitated with anti-AKT Ab or control rabbit IgG, and submitted to western blotting with anti-GILZ Ab. After stripping, membranes were reblotted with anti-total AKT mouse Ab. Blots: one representative experiment of three.

To study the effect of GILZ on cell proliferation in epithelial ovarian cancer, we generated BG-1 clones that stably and strongly express GILZ (pGILZ). As a control BG-1 cells were stably transfected with an empty vector (CTRL). pGILZ and CTRL clones were randomly selected for further experiments. The GILZ protein content was significantly higher in pGILZ clones than CTRL clones (Figure 3A). We then compared their spontaneous cell proliferation: it was significantly higher in pGILZ clones (*P *< 0.01) (Figure 3B). To confirm that GILZ overexpression increased the proliferation rate, CTRL and pGILZ clones were seeded at equal densities, and viable cells were counted over a 4-day period. Cells overexpressing GILZ grew faster than CTRL cells (Figure 3C). There was no difference in spontaneous apoptosis between pGILZ and CTRL clones [see Additional file 1].

We next investigated whether over-expression of GILZ affected AKT activation. p-AKT, currently the active form of AKT, was more abundant in pGILZ clones than in CTRL clones, whereas the status of phospho-ERK 1/2 remained unchanged (Figure 3D). In parallel, AKT activity was higher in pGILZ clones than in CTRL clones as assessed by testing for phosphorylation of glycogen synthase kinase (GSK-3α/β), a downstream target of AKT (Figure 3E). Thus GILZ overexpression induced an increase in p-AKT and an enhancement of AKT activity. AKT-binding proteins may cause structural changes and phosphorylations that activate AKT [27,28]. We also revealed the presence of GILZ-AKT complexes in BG-1 cells using immunoprecipitation experiments (Figure 3F).

### GILZ silencing reduces cell proliferation and AKT phosphorylation in BG-1 cells

**Figure 4:**
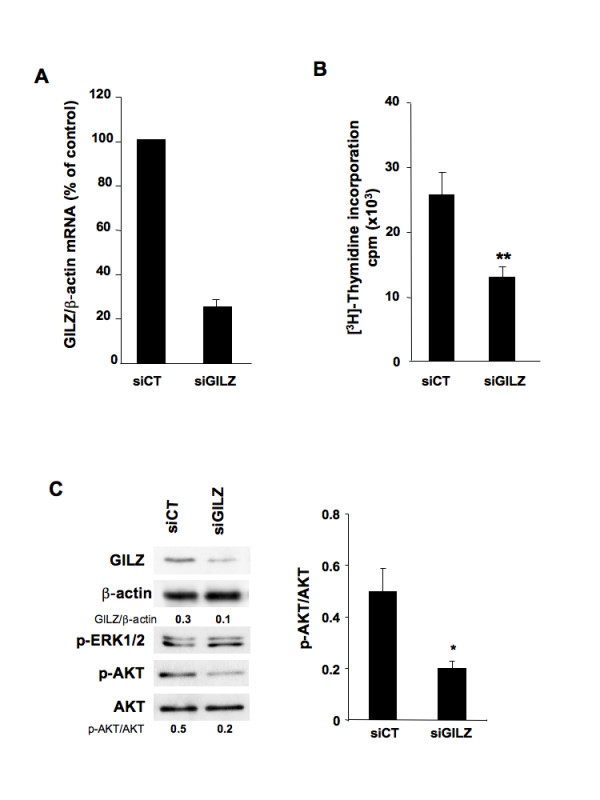
**GILZ down-regulation reduces cell proliferation and AKT phosphorylation in BG-1 cells**. BG-1 cells were transfected with 4 μg of control (siCT) or GILZ-specific (siGILZ) siRNA. (A) GILZ mRNA assayed by real-time RT-PCR and normalized to β-actin mRNA, 48 h after transfection. Results expressed as percentage of control from three independent experiments, mean ± SE. (B) Cell proliferation assayed by [^3^H]-thymidine incorporation 48 h after transfection with siRNA, mean ± SD, unpaired Student's t test was used for comparisons, **P < 0.01. (C) Left: total protein lysates were analyzed by western blotting using specific Abs. Blots: one representative experiment of three. Right: p-AKT expression levels were quantified by densitometric analysis and normalized to the signal for total AKT. Histogram represents mean ± SE of three experiments. Unpaired Student's t test was used for comparisons, *P < 0.05.

We studied the effects of knocking down *GILZ *mRNAs in BG-1 cells on cell proliferation and AKT activation. Real-time PCR and western blot analyses revealed that siRNA duplexes efficiently and specifically inhibited the expression of GILZ (mRNA and protein abundance) more than 75% lower than in cells treated with irrelevant control siRNA (Figure 4A).

Silencing *GILZ *gene expression led to a marked inhibition of cell proliferation and AKT phosphorylation, without changing phospho-ERK1/2 status (Figure 4, B and 4C).

Down regulation of GILZ expression in OVCAR3 cells, an ovarian cancer cell line that contains high amount of GILZ, also resulted in a decrease of cell proliferation [see Additional file 2]. These various findings reveal a previously unappreciated role of GILZ in the regulation of proliferation and AKT activation.

### GILZ controls p21 and cyclin D1 expression

**Figure 5:**
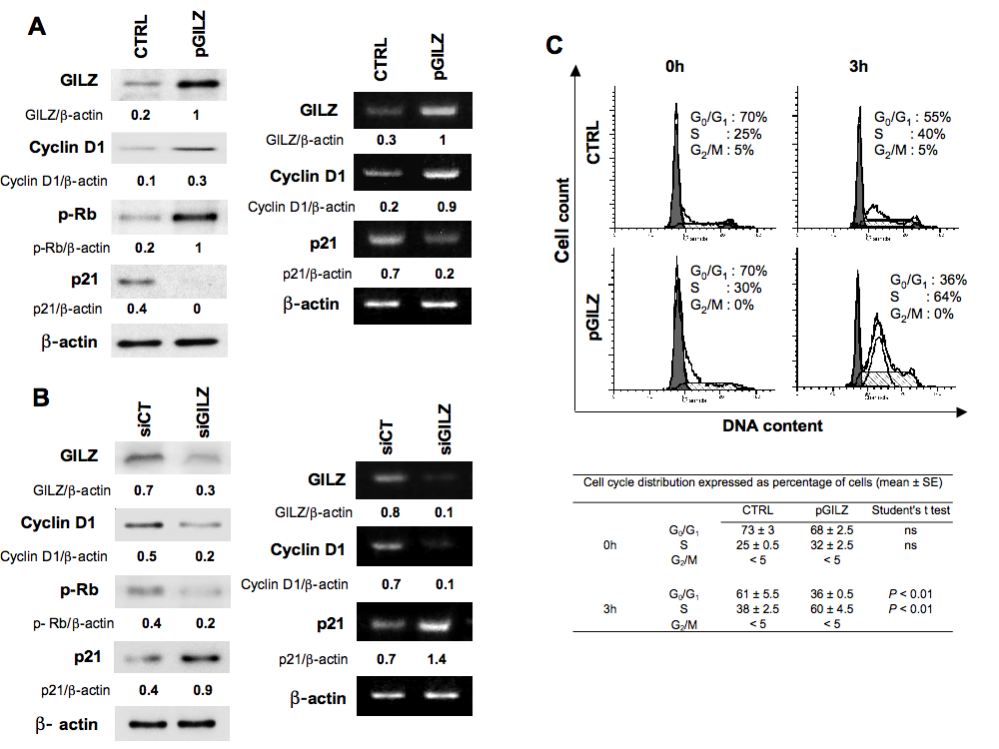
**GILZ controls cyclin D1 and p21 expression**. (A) Left: cyclin D1, phosphorylated retinoblastoma (p-Rb) and p21 were measured by western blot in CTRL and in pGILZ clones. β-actin was used as a loading control. Right: the steady-state levels of cyclin D1 and p21 mRNAs were assayed by semi-quantitative RT-PCR in CTRL and in pGILZ clones; β-actin was used as a loading control. (B) *p21 *and *cyclin D1 *gene products were measured by western blot (left) or by semi-quantitative RT-PCR (right) 48 h after BG-1 cells were transfected with 4 μg of control (siCT) or GILZ-specific (siGILZ) siRNA; β-actin was used as a loading control. Blots: one representative experiment of three. (C) Top: CTRL and pGILZ clones were synchronized by double thymidine block. Following removal of the block, cells were analyzed for DNA content by PI staining and cell cycle distribution was analyzed by flow cytometry at various time points; the percentages of cells in different cycle phases were determined by ModFit Cell Cycle Analysis software. Data are representative of three independent experiments. Bottom:Percentage of cells in each phase of the cell cycle. Results from three independent experiments (mean ± SE). Unpaired Student's t test was used for comparisons.

The cyclin-dependent kinase inhibitor p21 and cyclin D1 are two AKT targeted proteins that negatively (p21) and positively (cyclin D1) control cell cycle progression and proliferation [29]. Cyclin D1 activates cyclin-dependent-kinases (CDK4/6), leading to phosphorylation of retinoblastoma (Rb) with the resulting promotion of cell cycle progression [30]. We found that the overexpression of GILZ caused the up-regulation of cyclin D1 (mRNA and protein) and increased the amount of phosphorylated Rb (p-Rb); in contrast, p21 was down-regulated (Figure 5A). At the opposite, down regulation of GILZ resulted in decreased amount of *cyclin D1 *gene products (mRNA and protein) and p-Rb whereas those of *p21 *increased (Figure 5B). Thus the effects of down regulation of GILZ mirrored those of overexpression.

GILZ caused changes in p21 and cyclin D1 expression in such a way that increases in GILZ expression would accelerate cell cycle progression. To confirm this prediction we analyzed the cell cycle distribution of synchronized cells following removal of the thymidine block. We found that pGILZ cells entered S phase earlier than CTRL cells (Figure 5C).

### AKT activation contributes to BG-1 cell proliferation

**Figure 6:**
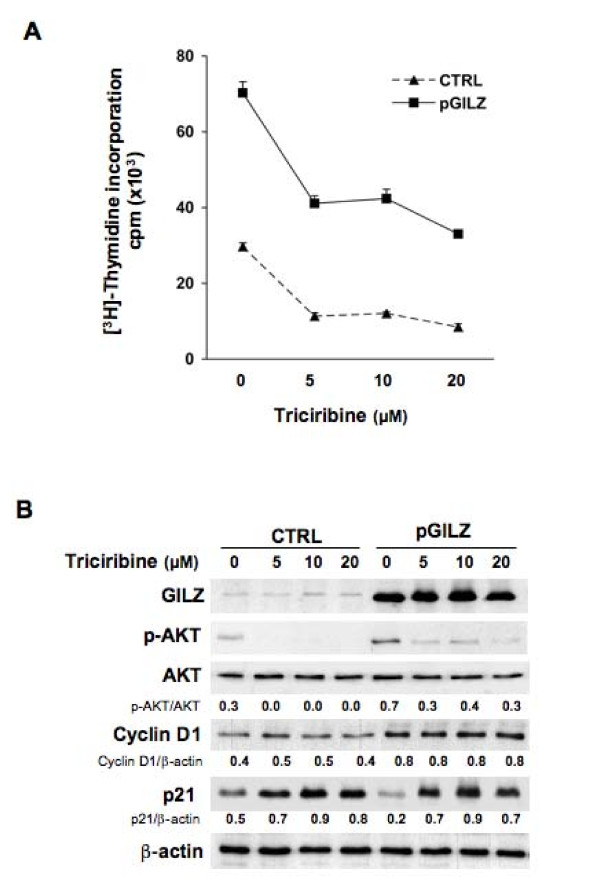
**AKT activation is required for BG-1 cell proliferation**. (A) Cell proliferation was measured by [^3^H]-thymidine incorporation in CTRL and pGILZ clones treated with indicated Triciribine doses for 24 h. Results are means ± SE of 3 independent experiments. (B) western blots of total protein lysates from CTRL or pGILZ clones treated with indicated Triciribine doses for 24 h. Membranes were probed with specific antibodies. The expression of GILZ, p-AKT, cyclin D1 and p21 proteins were quantified by densitometric analysis and normalized to β-actin or total AKT; results are expressed as GILZ/β-actin, p-AKT/AKT, cyclin D1/β-actin or p21/β-actin ratios. Blots: one representative experiment of three.

To investigate whether AKT activation is required for control of BG-1 cell proliferation, we used Triciribine, a specific pharmacological inhibitor of AKT phosphorylation. Triciribine treatment (5-20 μM) reduced p-AKT levels and in parallel decreased spontaneous proliferation of pGILZ and CTRL clones (Figure 6A). These findings indicate that AKT phosphorylation contributes to BG-1 cell proliferation. Further, Triciribine also caused an up-regulation of p21 expression in both CTRL and pGILZ clones. Interestingly, cyclin D1 expression remained unchanged. In addition, GILZ levels remained unchanged suggesting that p-AKT inhibition did not significantly affect GILZ expression (Figure 6B).

These various experiments show that AKT activation controls the expression level of p21 and contributes to cell proliferation in BG-1 cells. The enhancement of AKT activation by GILZ therefore accounts for GILZ effect, at least in part, on cell proliferation. In contrast, the enhancement of cyclin D1 promoted by GILZ is disconnected of its action on AKT.

## Discussion

The effects of GILZ have been mostly described in immune cells, particularly T-lymphocytes [17] or dendritic cells [10]. The role of GILZ in cancer is still poorly understood and most relevant work has been done in cell lines [19-22]. Here, we identified GILZ as a significant factor in the control of tumor cell proliferation in EOC.

This is the first report of the constitutive expression of GILZ in ovarian tumor specimens from patients with invasive ovarian carcinoma. Epithelial cells from malignant ascites, tumor specimens, and the ovarian cancer cell lines SKOV-3, OVCAR-3 and BG-1, all contain GILZ with a molecular weight of 17 kDa which is the original variant described by Riccardi and co-workers in 1997 [5].

Although contrasting views of the origin and histogenesis of EOC have been proposed, the epithelium that lines the ovarian surface is traditionally considered to be the most common origin of the neoplastic transformation [1,31]. Here, we did not detect GILZ on the surface epithelium of normal ovaries or in benign tumors, whereas it was expressed in most of EOC specimens, suggesting that GILZ is a molecule associated with malignant processes in ovaries. Ovarian epithelial tumors generally display morphological heterogeneity that pathologists classify into serous, clear cell, endometrioid, and mucinous subtypes on the basis of histopathological examination. Each subtype is characterized by specific genetic risk factors, molecular features, and mRNA expression profiles [32-34], suggesting that ovarian carcinoma is a heterogeneous disease [35]. Despite this heterogeneity, GILZ was detected in all the well-defined histological types and appears to be widely expressed in EOCs, and not restricted to particular histological subtypes.

GILZ was clearly confined to the cytoplasm in ovarian tumor cells. The intensity of GILZ staining and the proportion of tumor cells that were stained for GILZ differed between tumor sections. We found that this uneven production of GILZ in EOC correlated with the expression levels of Ki-67 when all the tumors were considered and also when the serous group was only considered. These findings were further supported by *in vitro *data demonstrating that tumor cell proliferation is regulated by GILZ expression level. Along with Ki-67, GILZ correlated with p-AKT, commonly used to characterize malignant tumor cells [23,24]. These findings were supported by *in vitro *data demonstrating that GILZ enhances p-AKT level and AKT activity. The PI3K/AKT pathway transmits mitogenic signals and controls cell cycle progression in ovarian cancer cells [26]. We found that p-AKT, the active form of AKT, accumulated in strongly positive GILZ tumor specimens. Further, up and down regulation of GILZ in BG-1 cells grown *in vitro *promoted parallel changes in the cellular abundance of p-AKT and in cell proliferation. In contrast, there was no feed back control of GILZ expression by p-AKT, unlike what has recently been reported in multiple myeloma [19]. AKT is frequently hyperactivated in EOC and contributes to the pathogenesis of ovarian cancer [25,36,37]. However, little is known about intracellular molecules that control AKT activation in tumor cells. Protein-protein interactions between GILZ and Raf and between GILZ and Ras have been reported in primary spleen T Lymphocytes and thymocytes [15,38]. As a consequence, GILZ inhibits downstream AKT cascades leading to antiproliferative effects in these cells. In contrast, our data are consistent with a model in which GILZ activates AKT and promotes cell proliferation. These findings probably reflect the large spectrum of GILZ actions and how they may differ substantially according to cell type and physio-pathological conditions. We also reveal the presence of GILZ-AKT complexes in BG-1 cells, suggesting that GILZ may be a novel partner of AKT. AKT-interacting proteins that bind to different functional domains have been widely reported [27]. They cause phosphorylations and/or structural changes that activate AKT and lock it in an active conformation. Our findings suggest that GILZ may provide intrinsic signals for AKT activation in the absence of external stimulation. Further studies will be needed to determine the precise molecular mechanisms underlying GILZ/AKT interaction.

Most of the G1-S regulators which control the G1-S transition, a crucial step in cell cycle progression, play also an important role in the tumor progression. Cyclin D1 is a positive regulator of progression through the G1 phase of the cell cycle. The transition to S phase is triggered by the activation of the cyclin D/CDK complex, which phosphorylates Rb, a well known regulator of cell proliferation [30]. At the opposite, p21, a universal CDK inhibitor, prevents cell cycle progression by acting at checkpoint G1 that causes sustained G1 blockade [29]. Importantly, we reveal that GILZ increases cyclin D1 expression and the amount of p-Rb, the essential substrate of cyclin D-CDK4/6 complex, whereas at the opposite it decreases p21 expression. All these effects that have never been reported before, are consistent with GILZ action on S-phase entry.

Using Triciribine, a pharmacological inhibitor of AKT activation, we reveal that BG-1 cell proliferation depends on AKT phosphorylation. In the same time we reveal that p21 which is negatively regulated by GILZ, is also reduced by AKT activation. This is consistent with a possible control of p21 expression by AKT as previously reported in various cell types [39]. Thus, GILZ-mediated enhancement of AKT activity may contribute to decrease p21 and to promote cell proliferation. In contrast, AKT is not required for cyclin D1 up regulation in BG-1 cells unlike what has been reported up to now in other cell types [40,41]. Possibly, GILZ may directly control the transcriptional activity of cyclin D1 as already demonstrated for other molecules [42].

## Conclusion

Few studies have identified particular molecules and their roles in the molecular mechanisms of tumor progression in EOC. Here, we report a previously unsuspected and important role for GILZ in the control of tumor cell proliferation in EOC. Our findings were supported by parallel and complementary data from tumor specimens and work with the BG-1 cellular model. They demonstrate that, in EOC, GILZ increases tumor cell proliferation, activates AKT, down-regulates p21 and promotes cyclin D1 expression; all these molecules are involved in the progression of malignant tumors and their deregulations are often associated to poor clinical outcome [43-46,25]. These findings highlight GILZ as a potential key molecule in EOC.

## Materials and methods

### Tissue samples

Approval was obtained from the ethics commission of the Antoine Béclère Hospital (Clamart, France) for all analyses of tumor material from clinical samples and from archival material from patients with a diagnosis of invasive ovarian carcinoma. Immunohistochemical examination of GILZ, phosphorylated protein kinase B/AKT (p-AKT) and Ki-67 proliferation index was performed retrospectively on tissue specimens of primary invasive ovarian carcinomas taken for routine diagnostic and therapeutic purposes from 50 patients who were treated surgically following a diagnosis of ovarian tumor at Antoine Béclère Hospital between 1998 and 2007. Clinical and pathological characteristics of the patients are detailed in Table 2. None of the patients had received neo-adjuvant chemotherapy before surgery. Clinical stage was assigned according to the International Federation of Gynecology and Obstetrics staging system (FIGO); histological subtypes and grades were assigned according to the criteria of the World Health Organization (WHO) classification [47].

**Table 2:** Clinical and histological parameters of patients

	**Patient number**	**Median**	**Percentage**	**Min-Max**
***Clinical parameters***				
Invasive ovarian tumors	50			
Age (years)		57		[30-86]
***Histologic types***				
Serous	26		52%	
Endometrioid	8		16%	
Mucinous	9		18%	
Clear Cells	5		10%	
Undifferentiated	2		4%	
***FIGO stages***				
FIGO stages IA-IC	14 (11 IA + 3 IC)		28%	
FIGO stages IIA-IIC	4 (2 IIA + 2 IIC)		8%	
FIGO stages IIIA-IIIC	30 (2 IIIA + 7 IIIB + 21 IIIC)		60%	
FIGO stage IV	2 IV		4%	
***Histologic Grades***				
Grade 1	9		18%	
Grade 2	11		22%	
Grade 3	23		46%	
undetermined	7		14%	

### Immunohistochemistry

We tested for GILZ, Ki-67 and p-AKT in EOC samples. Paraffin-embedded tissue sections were cut from representative blocks of tumor biopsies and probed with the following antibodies by the avidin-biotin peroxidase method (LSAB kit, Dako-France, Trappes): GILZ polyclonal antibody (Ab) (Santa Cruz, Le Perray-en-Yvelines, France, 1:100), Ki-67 monoclonal (m)Ab (Dako, 1:50) and phospho-AKT(ser473) Ab (Cell Signaling, St quentin-en-Yvelines, 78053, France, 1:50) which recognizes only the phosphorylated form of AKT [25,48]. Antigens were unmasked by incubation in 10 mmol/L sodium citrate buffer (Dako) and heating at 90°C using a microwave oven. Tissues were counterstained with hematoxylin. Negative controls were done without the primary Ab. Immunochemical staining was simultaneously interpreted by two independent investigators without knowledge of the patients' clinicopathological outcome. Immunostaining for GILZ and for Ki-67 were scored on a seven-tiered scale as follows: negative (0), 1 (weak intensity), 2 (moderate intensity) or 3 (strong intensity) combined with the percentage of positive cells scored as 0 (0%), 1 (1-10%), 2 (10-50%), 3 (50-80%), 4 (>80%) as previously reported [49]. P-AKT immunoreactivity was scored as low versus high, because sections with high AKT kinase activity have been reported to immunoreact strongly with phospho-AKT (ser473) Ab, presumably reflecting overexpression of the PI3K/AKT pathway, whereas no or weak p-AKT immunostaining has been described in tumor samples without increased AKT activity [49,50].

### Tumor cell enrichment from ascites

Tumor cell enrichment was based on the expression of CD326, a human epithelial antigen also known as EpCAM, which is broadly expressed on cells of epithelial origin and derived tumor cells [51]. CD326+ cells were positively selected using autoMACs columns (Myltenyi Biotech, Paris, France) from ascites collected from patients diagnosed with EOC. The percentage of CD326+ cells in the positive fraction was more than 80% as assessed using a FACScan flow cytometer (BD Biosciences).

### Cell culture and reagents

BG-1 cells derived from a solid tumor tissue of a patient with stage III ovarian adenocarcinoma (a kind gift from Dr G. Lazennec, U844 Inserm Montpellier, France) were maintained in Dulbecco's modified minimum essential medium (DMEM) supplemented with 10% fetal bovine serum (FBS), 2 mmol/L L-glutamine, 0.1 mg/mL streptomycin and 100 U/mL penicillin (Invitrogen, Ilkirch, France). The human ovarian carcinoma cell lines SKOV-3 and OVCAR-3 (American Type Culture Collection) were maintained in RPMI 1640 medium containing 0.1 mg/mL streptomycin, 100 U/mL penicillin, 2 mmol/L L-glutamine and 10% FBS. Triciribine was purchased from Calbiochem (VWR International SAS, Fontenay-sous-bois, France).

### Generation of BG-1 clones stably overexpressing GILZ

BG-1 cells were transfected using jetPEI (Polyplus-transfection, France) according to the manufacturer's protocol, with the *GILZ*-encoding vector pcDNA3-GILZ or with the empty vector pcDNA3 as a control. Forty-eight hours after transfection, stably transfected cells were selected by a 2-week treatment with 500 μg/mL of Geneticin (G-418; Invitrogen) and cloned by limiting dilution. Clones were then screened for GILZ expression using quantitative real-time PCR and immunoblot assays. GILZ expression remained stable over 7 days in culture. We thus generated BG-1 clones that stably and strongly expressed GILZ, named pGILZ clones. BG-1 clones stably transfected with an empty vector, named CTRL clones, were used as control.

### GILZ silencing

Small interfering RNA (siRNAs) duplexes were synthesized and tested for specific inhibition of GILZ expression as described previously [10]. BG-1 cells (5 × 10^5 ^cells/well) were transfected with 4 μg/well GILZ siRNA (siGILZ) or control siRNA (siCT), purchased from Qiagen (Courtaboeuf, France), by the lipofectamine method using X-tremeGENE siRNA transfection reagent according to the manufacturer's instructions (Roche Diagnostics, Meylan, France). Transfection efficiency was between 80% and 90%, as assessed by using fluorescent random siRNA: siRNA AlexaFluor 488 (Qiagen).

### RT-PCR procedures

Total RNA was extracted using a RNeasy Mini kit (Qiagen). The RNA was transcribed into cDNA by reverse transcription with random hexamers (Roche Diagnostics) and Moloney murine leukemia virus (MMLV) reverse transcriptase (Invitrogen). GILZ mRNA was quantified by real-time PCR on a Light Cycler instrument (Roche Diagnostics) using the FastStart DNA Master SYBER Green kit (Roche Diagnostics) as described previously [9,10]. Values were normalized to those for β-actin mRNA and are thus expressed as the GILZ/β-actin ratio. *p21 *and *cyclin D1 *mRNAs were assayed by semi-quantitative RT-PCR as described previously [52]. The primers used were as following: GILZ (294 bp) sense 5'-TCTGCTTGGAGGGGATGTGG-3', antisense 5'-ACTTGTGGGGATTCGGGAGC-3'; Cyclin D1 (413 bp) sense 5'-TGCATGTTCGTGGCCTCTAA-3', antisense 5'-CAGTCCGGGTCACACTTGAT-3'; p21 (331 bp) sense 5'-CGACTGTGATGCGCTAATGG-3', antisense 5'-CCGTTTTCGACCCTGAGAG-3'; β-actin (237 bp) sense 5'-GGGTCAGAAGGATTCCTATG-3', antisense 5'-GGTCTCAAACATGATCTGGG-3'.

### [^3^H] thymidine uptake

Cells were seeded in triplicate on 96-well plates at a density of 1 × 10^4 ^cells/well in DMEM medium with 10% FBS. Twenty-four hours later, [^3^H] thymidine (0.5 μCi/well) (PerkinElmer, Boston, US) was added and the samples incubated overnight. The radioactivity incorporated was determined as described previously [52] and results are expressed as counts per minute (cpm).

### Western blot and immunoprecipitation

Cells (2 × 10^6^) were lyzed as described previously [17]. Equivalent amounts of proteins were separated by SDS-polyacrylamide gel electrophoresis (SDS-PAGE), transferred to nitrocellulose membranes (Hybond-ECL, Amersham, Saclay, France) and probed with rabbit polyclonal Abs recognizing GILZ (Santa Cruz), AKT phosphorylated at Ser^473^, total AKT, and ERK1/2 phosphorylated at Thr^202/204^, and retinoblastoma phosphorylated at Ser^807/811 ^(all from Cell Signaling). Anti-cyclin D1 and anti-p21 mAbs were purchased from Cell Signaling. Loading controls used goat anti-β-actin Ab from Santa Cruz. Primary Abs were visualized using HRP-conjugated anti-rabbit, anti-mouse and anti-goat IgG (Santa Cruz) and enhanced chemiluminescence detection (Amersham). ScanAnalysis software (Biosoft, Cambridge, United Kingdom) was used for densitometric analysis.

Total protein lysates from BG-1 clones were immunoprecipitated with polyclonal anti-AKT Ab (Santa Cruz) overnight and then the immune complexes were precipitated with protein G bound to sepharose beads (Sigma-Aldrich). The immunoprecipitates were immunoblotted with anti-GILZ Ab to investigate the presence of GILZ.

### In Vitro kinase assay

The nonradioactive AKT kinase assay kit was used according to the manufacturer's instructions (Cell Signaling). Immobilized AKT mAb was used to immunoprecipitate AKT from cell lysates and the samples subjected to an *in vitro *kinase assay using GSK-3 fusion protein as a substrate. Phosphorylation of GSK-3 was measured by western blotting using phosphorylated GSK-3α/β (Ser^21/9^) Ab and chemiluminescent detection.

### Cell-cycle analysis

BG-1 cells were synchronized by double thymidine block as described previously [53]. After releasing the block in DMEM-10% FBS, cell cycles were analyzed using propidium iodide (PI) staining and fluorescence was measured using a FACScan flow cytometer. Cell cycle profiles were analyzed by ModFit Cell Cycle Analysis software.

### Statistical analysis

StatEL statistical software (Adscience, Paris, France) was used. The Spearman test was used to analyze the relationship between GILZ and Ki-67 scores. The two-tailed unpaired Student's *t *test was used to compare two groups and the Kruskal Wallis test followed by Dunn's test was used to compare several groups. Fisher's exact test was used to compare the relationship between the expression levels of GILZ and Ki-67 and of GILZ and p-AKT. Significance was set at *P *< 0.05.

## Competing interests

The authors declare that they have no competing interests.

## Authors' contributions

NR conceived the ideas with VM, performed all the in vitro experiments, analyzed data and contributed to manuscript draft. FG carried out immunohistochemical staining of tissue slides, quantified immunostaining and contributed to data analysis. CT collected tumor specimens and clinical data from patients and contributed to data analysis. MP and DE have been involved in revising the manuscript critically. A B-V contributed to GILZ overexpression experiments. HF provided with ovarian tumor specimens. SP analyzed tissue slides as a pathologist and contributed to provide ovarian tumor specimens as head of anatomo-cytology department. KB contributed to manuscript draft and writing. VM conceived the ideas with NJ, coordinated the experiments, analyzed the data, completed statistical analyses and wrote the manuscript. All authors read and approved the final manuscript.

## Supplementary Material

Additional file 1 **Effects of GILZ overexpression on spontaneous apoptosis**. CTRL or pGILZ cells were cultured at equal density in medium with 10% FBS for 24 h, and then stained with annexin V-FITC and propidium iodide and analyzed by flow cytometry. There was no difference in spontaneous apoptosis between pGILZ and CTRL clones. *Bottom*, summary data from three independent experiments (mean ± SE). Click here for file

Additional file 2 **GILZ down-regulation reduces cell proliferation in OVCAR-3 cells**. OVCAR-3 cells were transfected with 4 μg of control (siCT) or GILZ-specific (siGILZ) siRNA. (A) GILZ mRNA assayed by real-time RT-PCR and normalized to β-actin mRNA, 48 h after transfection. Results expressed as percentage of control from three independent experiments; error bars represent SE. (B) Cell proliferation assayed by [^3^H]-thymidine incorporation 48 h after transfection with siRNA. Results are mean of three independent experiments; error bars indicate the SE. Click here for file
