# Supplementary figures and images for: Identification of glucocorticoid-induced leucine zipper as a key regulator of tumor cell proliferation in epithelial ovarian cancer
Source: Mol Cancer. 2009 Oct 8;8:83. doi: 10.1186/1476-4598-8-83 (PMC2763858; doi:10.1186/1476-4598-8-83)

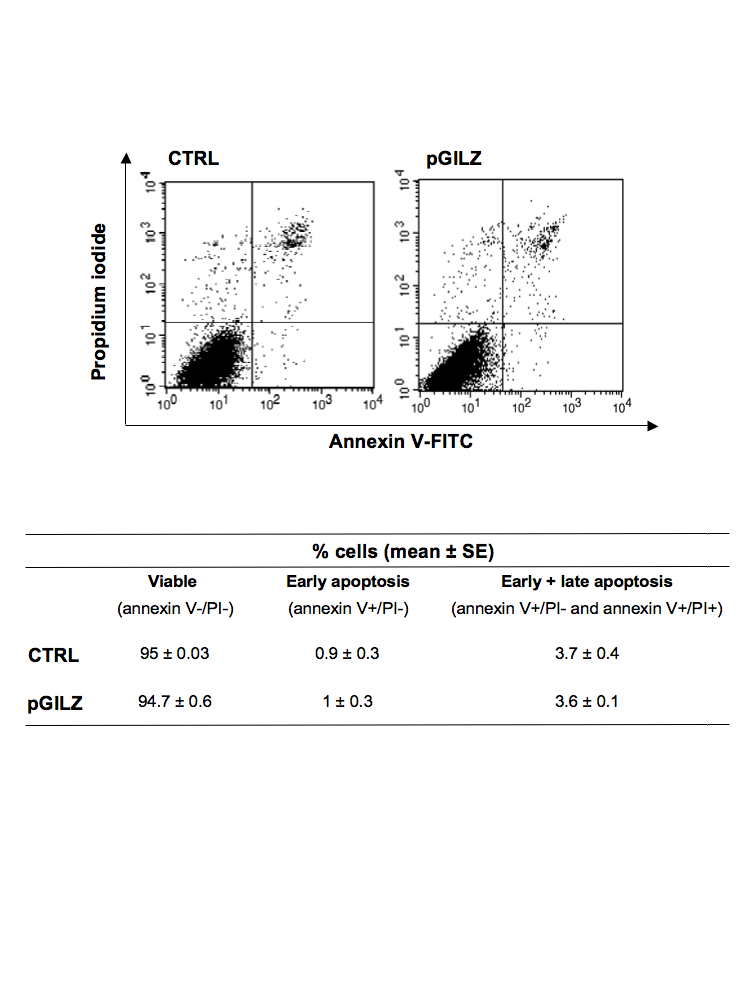

Supplement: Additional file 1 — Effects of GILZ overexpression on spontaneous apoptosis. CTRL or pGILZ cells were cultured at equal density in medium with 10% FBS for 24 h, and then stained with annexin V-FITC and propidium iodide and analyzed by flow cytometry. There was no difference in spontaneous apoptosis between pGILZ and CTRL clones. Bottom, summary data from three independent experiments (mean ± SE). [file 1476-4598-8-83-S1.TIFF]

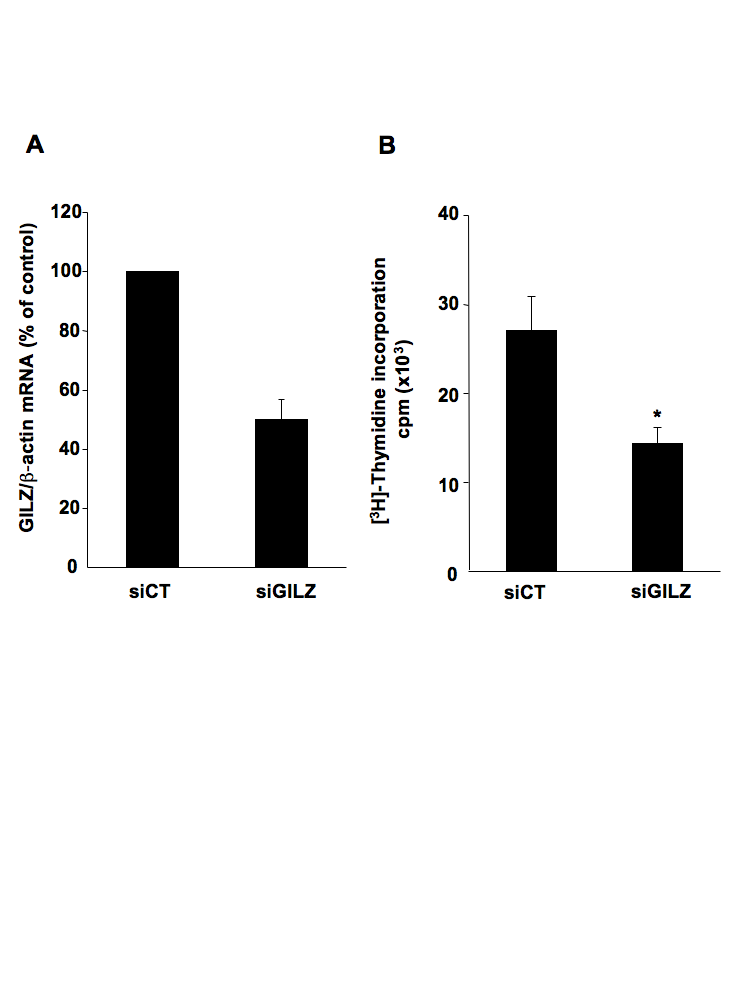

Supplement: Additional file 2 — GILZ down-regulation reduces cell proliferation in OVCAR-3 cells. OVCAR-3 cells were transfected with 4 μg of control (siCT) or GILZ-specific (siGILZ) siRNA. (A) GILZ mRNA assayed by real-time RT-PCR and normalized to β-actin mRNA, 48 h after transfection. Results expressed as percentage of control from three independent experiments; error bars represent SE. (B) Cell proliferation assayed by [3H]-thymidine incorporation 48 h after transfection with siRNA. Results are mean of three independent experiments; error bars indicate the SE. [file 1476-4598-8-83-S2.TIFF]
